# Clinical significance of serum CYFRA 21-1 in gastric cancer.

**DOI:** 10.1038/bjc.1996.288

**Published:** 1996-06

**Authors:** B. Nakata, Y. S. Chung, Y. Kato, M. Ogawa, Y. Ogawa, A. Inui, K. Maeda, T. Sawada, M. Sowa

**Affiliations:** First Department of Surgery, Osaka City University Medical School, Japan.

## Abstract

We studied the clinical significance of the soluble cytokeratin 19 fragment detected with monoclonal antibody CYFRA 21-1 in the sera of patients with histologically proven gastric cancer. Sera of 110 patients with gastric cancer were analysed for CYFRA 21-1 levels by a two-step sandwich enzyme immunoassay. There were no significant differences between CYFRA 21-1 levels and the histotype, depth of invasion or vessel invasion. However, CYFRA 21-1 was significantly higher in the presence of peritoneal metastases, liver metastases and extensive nodal involvement. When the positive cut-off value was defined as 5 ng ml-1, the CYFRA 21-1 in the stage IV and recurrent cases was 55.6% and 66.7%, respectively, which was as high as carcinoembryonic antigen (CEA) and greater than carbohydrate antigen 19-9 (CA 19-9). The positivities in stage I/II and III were zero and 5.9%, respectively, and false-positive rate in 76 patients with benign gastrointestinal disorders was 2.6%. There appeared to be no correlation between CYFRA 21-1 and CEA or CA 19-9. The patients with above 5 n ml-1 of CYFRA 21-1 had a significantly poorer prognosis. Multivariate analysis indicated that CYFRA 21-1 was an independent prognostic factor, while CEA and CA 19-9 failed to be of prognostic value. In conclusion, CYFRA 21-1 is a reliable tumour marker for gastic cancer in predicting very advanced cases, recurrence of the disease and overall poor prognosis.


© 1996 Stockton Press All rights reserved 0007-0920/96 $12.00

### Clinical significance of serum CYFRA 21-1 in gastric cancer

B Nakata, YS Chung, Y Kato, M Ogawa, Y Ogawa, A Inui, K Maeda, T Sawada and M Sowa

*First Department of Surgery, Osaka City University Medical School, Osaka 545, Japan.*

**Summary** We studied the clinical significance of the soluble cytokeratin 19 fragment detected with monoclonal antibody CYFRA 21-1 in the sera of patients with histologically proven gastric cancer. Sera of 110 patients with gastric cancer were analysed for CYFRA 21-1 levels by a two-step sandwich enzyme immunoassay. There were no significant differences between CYFRA 21-1 levels and the histotype, depth of invasion or vessel invasion. However, CYFRA 21-1 was significantly higher in the presence of peritoneal metastases, liver metastases and extensive nodal involvement. When the positive cut-off value was defined as 5 ng $ml^{-1}$, the CYFRA 21-1 in the stage IV and recurrent cases was 55.6% and 66.7%, respectively, which was as high as carcinoembryonic antigen (CEA) and greater than carbohydrate antigen 19-9 (CA 19-9). The positivities in stage I/II and III were zero and 5.9%, respectively, and false-positive rate in 76 patients with benign gastrointestinal disorders was 2.6%. There appeared to be no correlation between CYFRA 21-1 and CEA or CA 19-9. The patients with above 5 n $ml^{-1}$ of CYFRA 21-1 had a significantly poorer prognosis. Multivariate analysis indicated that CYFRA 21-1 was an independent prognostic factor, while CEA and CA 19-9 failed to be of prognostic value. In conclusion, CYFRA 21-1 is a reliable tumour marker for gastric cancer in predicting very advanced cases, recurrence of the disease and overall poor prognosis.

**Keywords:** CYFRA 21-1; gastric cancer; clinicopathological factor; prognosis

Many clinically useful epitopes of tumour-associated antigens belong to glycoproteins shedding from the cell surface, i.e. CEA, CA 19-9, sialyl Tn antigen and alpha-fetoprotein (AFP). CYFRA 21-1 is unique in that its epitope is from a polypeptide which is most likely released following cell death (Stieber *et al.*, 1993*a*). CYFRA 21-1, which recognises soluble cytokeratin 19 fragments (Bodenmüller *et al.*, 1992, 1994*a*), has been introduced as the most sensitive tumour marker for lung carcinomas, except for small-cell lung cancer (Pujol *et al.*, 1993; Stieber *et al.*, 1993*a,b*; van der Gaast *et al.*, 1994; Takada *et al.*, 1995). Aside from lung cancer, CYFRA 21-1 has been reported in uterine carcinomas (Ferdeghini *et al.*, 1993; Bonfrer *et al.*, 1994) and head and neck carcinomas (Doweck *et al.*, 1995). Little is known, however, about the clinical significance of serum CYFRA 21-1 titres in gastric cancer. In this study, the association between serum CYFRA 21-1 level and the clinicopathological features and prognosis in patients with gastric cancer was studied. Further, the clinical usefulness of CYFRA 21-1 as a tumour marker in gastric cancer was considered.

### Materials and methods

### *Patients*

The sera from 110 patients with gastric cancer was obtained between January and December 1992 at the First Department of Surgery, Osaka City University Medical School, and was measured for CYFRA 21-1. The patients consisted of 101 primary and 9 recurrent cases. All living patients were followed for more than 30 months. Additionally, we measured CYFRA 21-1 levels in the sera of 100 healthy individuals and in 76 patients with benign disorders of the gastrointestinal tract. The serum samples were stored at −70°C until assayed. Clinicopathological features and staging were classified according to the Japanese Classification of Gastric Carcinoma (1995). The survival period was defined as the time after the serum sample was taken until the day of death.

Correspondence: B Nakata, First Department of Surgery, Osaka City University Medical School, 1-5-7 Asahimachi, Abeno-ku, Osaka, 545 Japan
Received 22 September 1995; revised 13 December 1995; accepted 9 January 1996

### *Assay*

The measurement of CYFRA 21-1 was completed in a two-step sandwich enzyme immunoassay using the Enzymun-test kit for CYFRA 21-1 (Boehringer Mannheim, Mannheim, Germany) as previously described (Takada *et al.*, 1995). The kit was composed of two mouse monoclonal antibodies, Ks 19.1 and BM 19.21.

In addition, we also determined the carcinoembryonic antigen (CEA) levels as well as carbohydrate antigen 19-9 (CA 19-9) levels which are currently established tumour-associated antigens for gastric cancer. CEA and CA 19-9 were measured by counting immunoassay using commercially avialable kits (Ranream CEA, TOA Medical Electronics, Kobe, Japan; Ranream CA 19-9, Toray-Fuji Bionics, Tokyo, Japan) in conjunction with automated PAMIA-100 analyser (TOA Medical Electronics). The cut-off values of CEA and CA 19-9 recommended by the manufacturers were 6.5 ng $ml^{-1}$ and 37 U $ml^{-1}$ respectively.

### *Statistical assessment*

Statistical analysis was performed using a non-parametric method. The Mann-Whitney *U*-test was used for comparison of two independent groups. The Kruskal–Wallis one-way analysis was performed for multiple comparison tests. Correlation coefficients were assessed by simple linear regression analysis. Survival analysis of single variables was estimated by the Kaplan–Meier method and examined by the log-rank and Wilcoxon test. Multivariate analysis of survival was completed using the Cox proportional hazards model. The test results were regarded as significant if $P<0.05$.

### Results

### *Serum CYFRA 21-1 titre of the patients with primary gastric cancer*

In the patients with primary gastric cancer, the serum CYFRA 21-1 titre ranged from 0.4 to 110 ng $ml^{-1}$ with a median value of 1.6 ng $ml^{-1}$. The median value was significantly higher than that of healthy controls, although it was not significantly higher than that of patients with benign gastrointestinal disorders (Table I).

There was no significant difference among the serum

**Table I** Comparison of serum CYFRA 21-1 levels among the various subjects

| Subject | Number | CYFRA 21-1 ($mg\,ml^{-1}$) Median | Range |
|---|---|---|---|
| Primary gastric cancer | 101 | 1.6 | 0.4-110 |
| Recurrent gastric cancer | 9 | 15.0 | 3.3-36 |
| Local | 1 | 3.3 | |
| Peritoneal | 2 | 6.9 | 3.7-10 |
| Liver/lung/adrenal | 6 | 27.0 | 3.3-36 |
| Benign digestive disorder | 76 | 1.5 | 0.3-5.5 |
| Stomach | 13 | 1.6 | 0.6-2.3 |
| Intestinal | 9 | 1.1 | 0.5-1.7 |
| Liver (cirrhosis) | 9 | 2.1 | 1.1-5.5 |
| Gall bladder | 39 | 1.5 | 0.3-5.0 |
| Pancreas | 6 | 1.4 | 0.6-2.1 |
| Healthy controls | 100 | 1.1 | 0.5-9.5 |

CYFRA 21-1 titres of histological types by the Kruskal–Wallis test ($P=0.151$). Additionally, in regard to the depth of invasion of the gastric wall, venous invasion or lymphatic involvement, serum CYFRA 21-1 titre did not significantly differ by the Kruskal–Wallis test ($P=0.106$, 0.094 or 0.065, respectively).

The main sites of metastases for gastric cancer were the intraperitoneal cavity, liver and lymph nodes. Figure 1 shows serum CYFRA 21-1 titres according to the metastatic status. For peritoneal metastases, the serum CYFRA level was significantly elevated from P0 to P2 (Kruskal–Wallis test). The median value of serum CYFRA 21-1 for patients with peritoneal metastases (P1, P2) was significantly higher than those without peritoneal metastasis (P0) by the Mann–Whitney *U*-test (2.6 ng $ml^{-1}$ *vs* 1.5 ng $ml^{-1}$). The serum CYFRA 21-1 titre differed by the status of the liver metastases (Kruskal–Wallis test). Mann–Whitney *U*-test showed significant differences between the CYFRA 21-1 median values of patients with and without liver metastases (8.1 ng $ml^{-1}$ *vs* 1.5 ng $ml^{-1}$). As for lymph node involvement, CYFRA 21-1 levels differed significantly according to the metastatic status (Kruskal–Wallis test). Patients with marked lymph node metastases (n3, n4) had significantly higher serum CYFRA 21-1 levels than patients from n0 to n2 (Mann–Whitney *U*-test; median value, 2.5 ng $ml^{-1}$ *vs* 1.5 ng $ml^{-1}$). Figure 2 shows how serum CYFRA 21-1 levels vary according to staging. There was no significant difference among the CYFRA 21-1 values at each stage by the Kruskal–Wallis test. The median value of CYFRA 21-1 for stage IV disease was significantly higher than that below stage III (Mann–Whitney *U*-test; median value, 6.2 ng $ml^{-1}$ *vs* 1.5 ng $ml^{-1}$).

*Serum CYFRA 21-1 titre of the patients with recurrent gastric cancer*

The patients with recurrent gastric cancer had significantly higher serum CYFRA 21-1 levels compared with healthy controls, patients with benign gastrointestinal diseases and patients with primary gastric cancer (Table I, Mann–Whitney *U*-test, $P<0.0001$).

*Prognosis and the serum CYFRA 21-1 titre*

During the survey period of the primary cases, 82 patients survived without recurrence, two patients survived with recurrence of peritoneal metastases and 16 patients died secondary to recurrence. One patient whose serum CYFRA 21-1 titre was 110 ng $ml^{-1}$ died of disseminated intravascular coagulation (DIC) following surgery. The CYFRA 21-1 median serum level in those patients who survived was 1.5 ng $ml^{-1}$, while the level in those who died was significantly higher (median value of 4.1 ng $ml^{-1}$). All patients with recurrent disease were dead in 1–4 months after the serum samples were acquired. Gastric cancer patients with serum CYFRA 21-1 levels over 5 ng $ml^{-1}$ proved to have a significantly shorter overall survival than those with lower serum levels (Figure 3).

**Figure 1** Serum CYFRA 21-1 distribution according to the status of peritoneal metastases, liver metastases and lymph node metastases. The vertical line, median value; the column, interquartile range. Peritoneal metastases: P0, no peritoneal metastasis; P1, metastases to the adjacent peritoneum but not the distant peritoneum; P2, a few metastases to the distant peritoneum; P3, numerous metastases to the distant peritoneum. Liver metastases: H0, no liver metastases; H1, metastases limited to one lobe; H3, numerous metastases to both lobes. Lymph node metastases: n0, no evidence of lymph node metastases; n1–n4, metastases to group 1–4 lymph nodes, respectively, which are defined in the *Japanese Classification of Gastric Carcinoma*. (Japanese Research Society for Gastric Cancer, 1995).

*Comparison and correlation of CYFRA 21-1 with CEA and CA 19-9*

When the cut-off value of serum CYFRA 21-1 for gastric cancer was set at 5 ng $ml^{-1}$, the sensitivities of the cases in stage I/II and stage III were zero and 5.9% respectively. However, 55.6% of the cases in stage IV and 66.7% of recurrent cases could be detected. Whereas the sensitivity of serum CEA was 24.1% in stage I, it was similar to CYFRA 21-1 in stage IV and recurrent cases. The CA 19-9 levels were lower than CYFRA 21-1 in both stage IV and recurrent cases (Figure 4).

**Figure 2** Serum CYFRA 21-1 distribution according to stage. The horizontal line, median value.

**Figure 3** Probability of survival of the patients with gastric cancer in relation to their serum CYFRA 21-1 levels.

There appeared not to be any correlation between serum CYFRA 21-1 titres and serum CEA or CA 19-9 (correlation coefficients were 0.10 or 0.18 respectively). When CYFRA 21-1 was combined with CEA and CA 19-9, the true-positive rate increased to 66.7% in stage IV disease and in 88.9% of recurrent cases.

### *Serum CYFRA 21-1 titre and prognosis*

The Cox proportional hazards model was performed on five clinicopathological factors (peritoneal metastases, liver metastases, lymph node metastases, lymphatic invasion and venous invasion) and three tumour markers including CYFRA 21-1. For these tumour markers, CYFRA 21-1 alone was an independent factor which affected prognosis (Table II).

### Discussion

Cytokeratins comprise the intermediate filaments of the cytoskeleton of epithelial cells (Steinert and Roop, 1988).

**Figure 4** Comparison of the sensitivity of CYFRA 21-1, CEA and CA 19-9 for each stage and recurrence of gastric cancer. ■, CYFRA 21-1; □, CEA; ▨, CA 19-9.

Nineteen kinds of cytokeratins have been subdivided according to their position on a two-dimensional gel (Moll *et al.*, 1982; Debus *et al.*, 1984). There is a specific pattern of cytokeratin expression for each type of epithelial cell (Moll *et al.*, 1982; Broers *et al.*, 1988; Sundström *et al.*, 1989). Cytokeratin 19 exists in normal epithelial cells and their malignant counterparts, although it is not detected in hepatocytes or epidermis (Moll *et al.*, 1982). A newly established monoclonal antibody, CYFRA 21-1, has been shown to react exclusively with cytokeratin 19 (Bodenmüller *et al.*, 1994*b*). In addition, CYFRA 21-1 has been well documented as an excellent tumour marker for non-small-cell lung cancer. A few studies on CYFRA 21-1 for gastrointestinal cancer, however, reported limited clinical usefulness for its diagnostic use because of low sensitivity (Stieber *et al.*, 1993*a*, *b*).

In the current study, the serum CYFRA 21-1 titres of patients with primary gastric cancer were not significantly higher than those of benign gastrointestinal disorders. However, when serum CYFRA 21-1 was analysed by stage grouping, stage IV cases showed significantly higher levels than those of stage III and below. Recurrent cases also had high levels of serum CYFRA 21-1.

We further investigated factors which could potentially elevate CYFRA 21-1 levels. Histotype, depth of invasion or vessel invasion were not associated with serum CYFRA 21-1 levels. Conversely, peritoneal metastases, liver metastases and lymph node involvement may well be significant factors involved in serum CYFRA 21-1 elevation. Our data supports the assumption that serum CYFRA 21-1 titre might be associated with tumour bulk.

We defined the cut-off value as 5.0 ng $ml^{-1}$ to select for

**Table II** Multivariate analysis with the Cox proportional hazards model

| *Variable* | *Coefficient* | *Standard error* | P-*value* | *95%CI* | *Hazard ratio* |
|---|---|---|---|---|---|
| Peritoneal metastases | −0.269 | 0.422 | 0.524 | 0.334–1.748 | 0.764 |
| Liver metastases | 1.626 | 0.615 | 0.008 | 1.522–16.972 | 5.082 |
| Lymph node metastases | 1.573 | 0.374 | <0.0001 | 2.319–10.033 | 4.823 |
| Lymphatic invasion | 1.211 | 0.496 | 0.015 | 1.269–8.881 | 3.357 |
| Venous invasion | −1.624 | 0.558 | 0.004 | 0.066–0.589 | 0.197 |
| CYFRA 21-1 | 0.051 | 0.023 | 0.027 | 1.006–1.100 | 1.052 |
| CA 19-9 | 0.003 | 0.003 | 0.389 | 0.997–1.009 | 1.003 |
| CEA | −0.0001 | 0.0008 | 0.905 | 0.998–1.002 | 1.000 |

advanced cases of gastric cancer from the distribution of serum CYFRA 21-1 titres in our series (Figure 3). This value is a little higher than the cut-off level of 3.5 ng $ml^{-1}$ recommended by the Japan CYFRA research group to distinguish between benign and malignant lung disease (Sugama *et al.*, 1994). When the cut-off value of 5.0 ng $ml^{-1}$ was employed, the sensitivities of serum CYFRA 21-1 in patients with stage IV and recurrent gastric cancer were quite high, although overall sensitivity for primary gastric cancer was 6.0%. This phenomenon might be related to the release mechanism of cytokeratins. These levels should appear elevated in serum after the tumour grows to an appropriate size with subsequent necrosis. The false-positive rate in benign gastrointestinal disorders was 2.6% (2 of 76 cases). In benign disease of the gastrointestinal tract, the patients with the liver cirrhosis showed relatively high serum CYFRA 21-1 levels coinciding with other reports (Molina *et al.*, 1994). Accordingly, this must be taken into account when evaluating patients with gastric cancer complicated with liver cirrhosis.

Moreover, the patient whose serum CYFRA 21-1 level was above 5 ng $ml^{-1}$ overall had a significantly poorer prognosis. Multivariate analysis also showed that serum CYFRA 21-1 could function as an independent prognostic determinant, although the hazard ratio was 1.052 indicating it did not add strong prognostic information.

There was no correlation between CYFRA 21-1 and CEA or CA 19-9 levels, indicating the potential usefulness in combining these markers to detect advanced or recurrent gastric cancer. Similar results were reported in lung cancer studies (Sugama *et al.*, 1994; van der Gaast *et al.*, 1994). Indeed, the combination of these markers improved the sensitivity.

Finally, the measurement of serum CYFRA 21-1 in patients with gastric cancer has proven to be clinically useful for selecting patients with very advanced disease, monitoring for tumour recurrence and predicting the overall prognosis.

### References

BODENMÜLLER H, BANAUCH D, OFENLOCH-HÄHNLE B, JAWOREK D AND DESSAUER A. (1992). Technical evaluation of a new automated tumor marker assay: The enzymun-test Cyfra 21.1, In *Tumor associated Antigens, Oncogenes, Receptors, Cytokines in Tumor Diagnosis and Therapy at the Beginning of the Nineties. Cancer of the Breast, State and Trends in Diagnosis and Therapy*, Klapdor R (ed.) pp. 137–138. Zuckschwerdt: Munich.

BODENMÜLLER H, DONIE F, KAUFMANN M AND BANAUCH D. (1994*a*). The tumor markers TPA, TPS, $TPA_{CYK}$ and CYFRA21-1 react differently with the keratins 8, 18 and 19. *Int. J. Biol. Markers*, **9**, 70–74.

BODENMÜLLER H, OFENLOCH-HÄHNLE B, LANE EB, DESSUAR A, BOTTGER V AND DONIE F. (1994*b*). Lung cancer-associated keratin 19 fragments: development and biochemical characterization of the new serum assay Enzymun-Test CYFRA 21-1. *Int. J. Biol. Markers*, **9**, 75–81.

BONFRER JM, GAARENSTROOM KN, KENTER GG, KORSE CM, HART AA, GALLEE MP, HELMERHORST TJ AND KENEMANS P. (1994). Prognostic significance of serum fragments of cytokeratin 19 measured by Cyfra 21-1 in cervical cancer. *Gynecol. Oncol.*, **55**, 371–375.

BROERS JL, RAMAEKERS FC, ROT MK, OOSTENDORF T, HUYSMANS A, VAN MUIJEN GN, WAGENAAR SS AND VOOIJS GP. (1988). Cytokeratins in different types of human lung cancer as monitored by chain-specific monoclonal antibodies. *Cancer Res.*, **48**, 3221–3229.

DEBUS E, MOLL R, FRANKE WW, WEBER K AND OSBORN M. (1984). Immunohistochemical distinction of human carcinomas by cytokeratin typing with monoclonal antibodies. *Am. J. Pathol.*, **114**, 121–130.

DOWECK I, BARAK M, GREENBERG E, URI N, KELLNER J, LURIE M AND GRUENER N. (1995). Cyfra 21-1. A new potential tumor marker for squamous cell carcinoma of head and neck. *Arch. Otolaryngol. Head Neck Surg.*, **121**, 177–181.

FERDEGHINI M, GADDUCCI A, ANNICCHIARICO C, PRONTERA C, MALAGNINO G, CASTELLANI C, FACCHINI V AND BIANCHI R. (1993). Serum CYFRA 21-1 assay in squamous cell carcinoma of the cervix. *Anticancer Res.*, **13**, 1841–1844.

JAPANESE RESEARCH SOCIETY FOR GASTRIC CANCER. (1995). *Japanese Classification of Gastric Carcinoma*. First English edition. Kanehara: Tokyo.

MOLINA R, AGUSTI C, FILELLA X, JO J, JOSEPH J, GIMÉNEZ N AND BALLESTA AM. (1994). Study of a new tumor marker, CYFRA 21-1, in malignant and nonmalignant diseases. *Tumor Biol.*, **15**, 318–325.

MOLL R, FRANKE WW, SCHILLER DL, GEIBER B AND KREPLER R. (1982). The catalog of human cytokeratins: patterns of expression in normal epithelia, tumors and cultured cells. *Cell*, **31**, 11–24.

PUJOL J-L, GRENIER J, DAURÈS J-P, DAVER A, PUJOL H AND MICHEL F-B. (1993). Serum fragment of cytokeratin subunit 19 measured by CYFRA 21-1 immunoradiometric assay as a marker of lung cancer. *Cancer Res.*, **53**, 61–66.

STEINERT PM AND ROOP DR. (1988). Molecular and cellular biology of intermediate filaments. *Ann. Rev. Biochem.*, **57**, 593–625.

STIEBER P, BODENMÜLLER H, BANAUCH D, HASHOLZNER U, DESSAUER A, OFENLOCH-HÄHNLE B, JAWOREK D AND FATEH-MOGHADAM A. (1993*a*). Cytokeratin 19 fragments: a new marker for non-small-cell lung cancer. *Clin. Biochem.*, **26**, 301–304.

STIEBER P, HASHOLZNER U, BODENMÜLLER H, NAGEL D, SUNDER-PLASSMAN L, DIENEMANN H, MEIER W AND FATEH-MOGHADAM A. (1993*b*). CYFRA 21-1. A new marker in lung cancer. *Cancer*, **72**, 707–713.

SUGAMA Y, KITAMURA S, KAWAI T, OHKUBO A, HASEGAWA S, KURIYAMA T, KATO H, FUKUOKA M AND OHKAWA J. (1994). Clinical usefulness of CYFRA assay in diagnosing lung cancer: measurement of serum cytokeratin fragment. *Jpn. J. Cancer Res.*, **85**, 1178–1184.

SUNDSTRÖM BE, NATRATH WBJ AND STIGBRAND T. (1989). Diversity in immunoreactivity of tumor derived cytokeratin monoclonal antibodies. *Histochem. Cytochem.*, **37**, 1848–1854.

TAKADA M, MASUDA N, MATSUURA E, KUSUNOKI Y, MATUI K, NAKAGAWA K, YANA T, TUYUGUCHI I, OOHATA I AND FUKUOKA M. (1995). Measurement of cytokeratin 19 fragments as a marker of lung cancer by CYFRA 21-1 enzyme immunoassay. *Br. J. Cancer*, **71**, 160–165.

VAN DER GAAST A, SCHOENMAKERS CHH, KOK TC, BLIJENBERG BG, CORNILLIE F AND SPLINTER TAW. (1994). Evaluation of a new tumor marker in patients with non-small-cell lung cancer: Cyfra 21-1. *Br. J. Cancer*, **69**, 525–528.


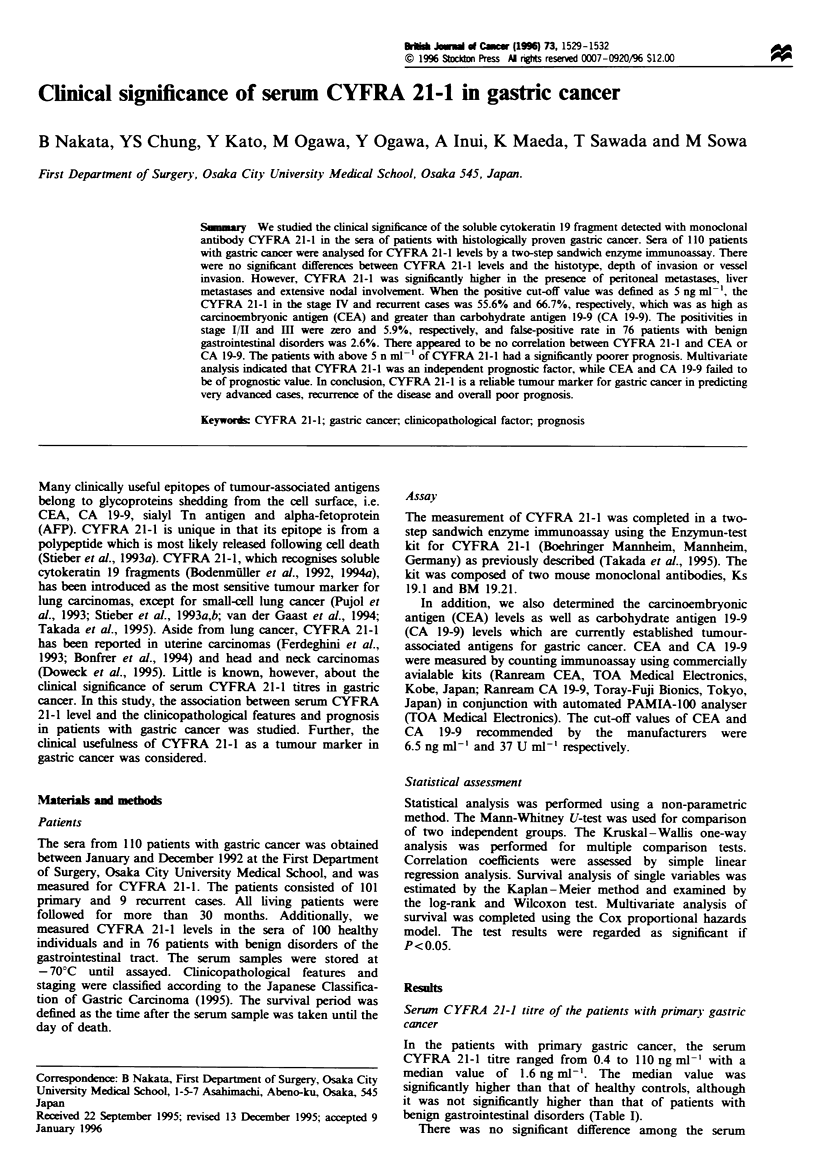

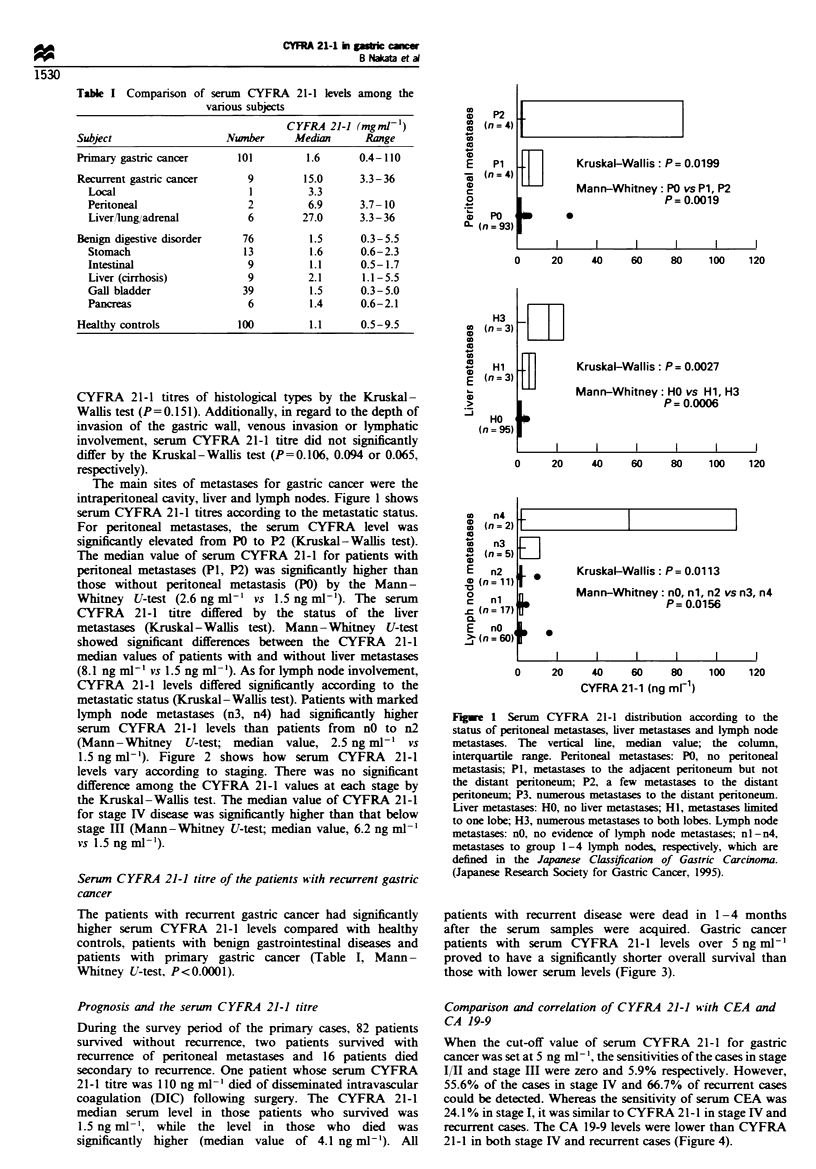

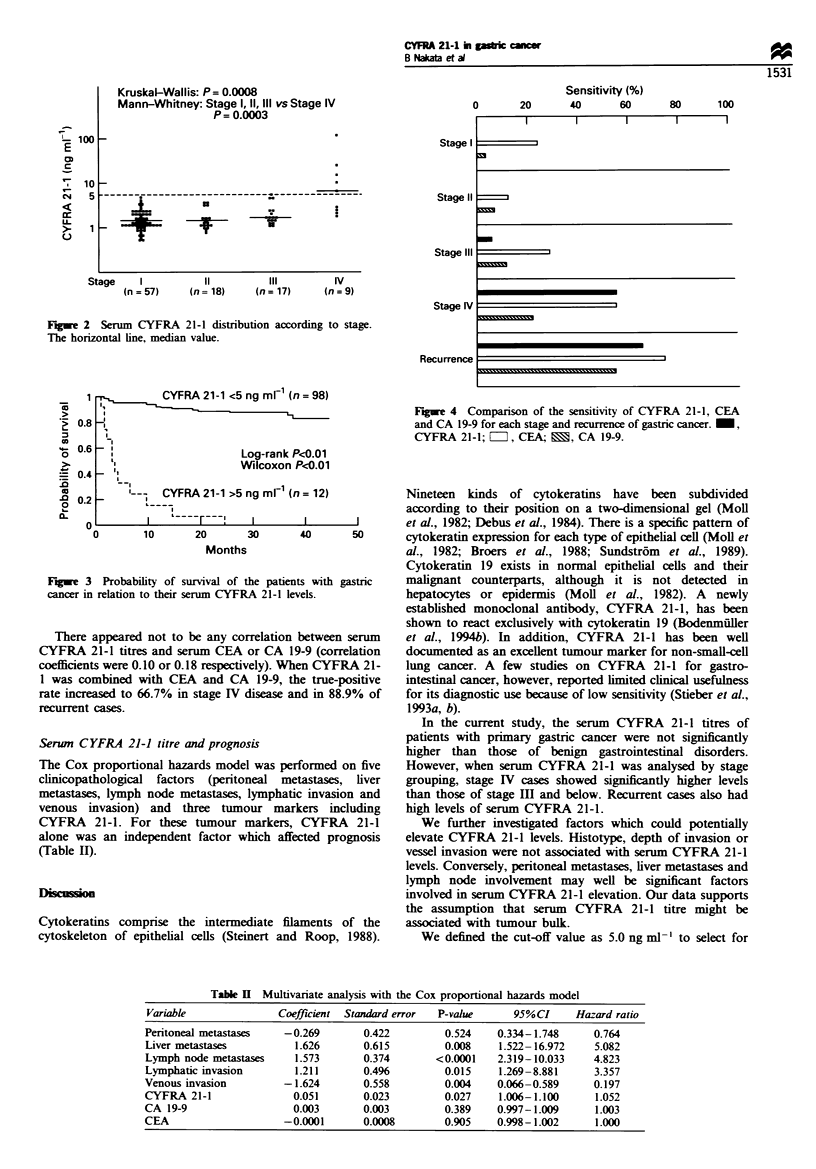

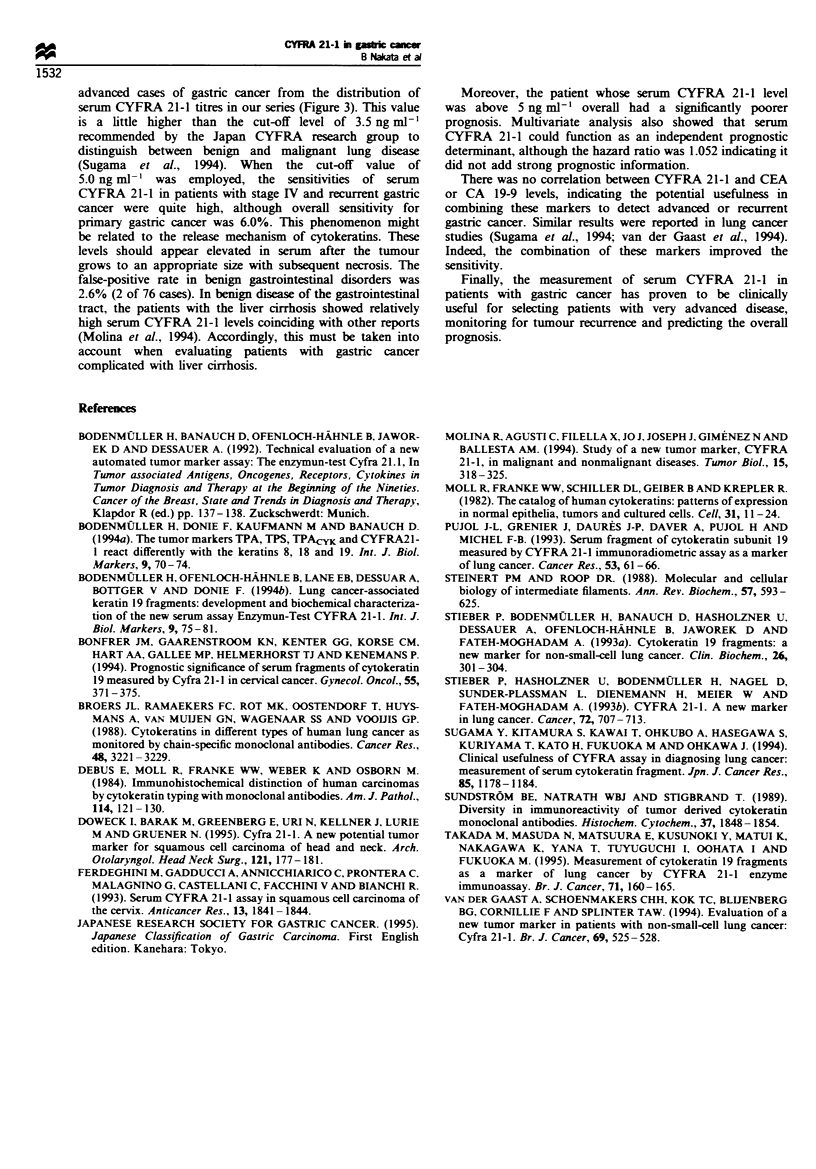


## References

[OCR_00584] Bodenmüller H., Donié F., Kaufmann M., Banauch D. (1994). The tumor markers TPA, TPS, TPACYK and CYFRA 21-1 react differently with the keratins 8, 18 and 19.. Int J Biol Markers.

[OCR_00588] Bodenmüller H., Ofenloch-Hähnle B., Lane E. B., Dessauer A., Böttger V., Donié F. (1994). Lung cancer-associated keratin 19 fragments: development and biochemical characterisation of the new serum assay Enzymun-Test CYFRA 21-1.. Int J Biol Markers.

[OCR_00595] Bonfrer J. M., Gaarenstroom K. N., Kenter G. G., Korse C. M., Hart A. A., Gallee M. P., Helmerhorst T. J., Kenemans P. (1994). Prognostic significance of serum fragments of cytokeratin 19 measured by Cyfra 21-1 in cervical cancer.. Gynecol Oncol.

[OCR_00605] Broers J. L., Ramaekers F. C., Rot M. K., Oostendorp T., Huysmans A., van Muijen G. N., Wagenaar S. S., Vooijs G. P. (1988). Cytokeratins in different types of human lung cancer as monitored by chain-specific monoclonal antibodies.. Cancer Res.

[OCR_00611] Debus E., Moll R., Franke W. W., Weber K., Osborn M. (1984). Immunohistochemical distinction of human carcinomas by cytokeratin typing with monoclonal antibodies.. Am J Pathol.

[OCR_00618] Doweck I., Barak M., Greenberg E., Uri N., Kellner J., Lurie M., Gruener N. (1995). Cyfra 21-1. A new potential tumor marker for squamous cell carcinoma of head and neck.. Arch Otolaryngol Head Neck Surg.

[OCR_00624] Ferdeghini M., Gadducci A., Annicchiarico C., Prontera C., Malagnino G., Castellani C., Facchini V., Bianchi R. (1993). Serum CYFRA 21-1 assay in squamous cell carcinoma of the cervix.. Anticancer Res.

[OCR_00635] Molina R., Agusti C., Filella X., Jo J., Joseph J., Giménez N., Ballesta A. M. (1994). Study of a new tumor marker, CYFRA 21-1, in malignant and nonmalignant diseases.. Tumour Biol.

[OCR_00638] Moll R., Franke W. W., Schiller D. L., Geiger B., Krepler R. (1982). The catalog of human cytokeratins: patterns of expression in normal epithelia, tumors and cultured cells.. Cell.

[OCR_00645] Pujol J. L., Grenier J., Daurès J. P., Daver A., Pujol H., Michel F. B. (1993). Serum fragment of cytokeratin subunit 19 measured by CYFRA 21-1 immunoradiometric assay as a marker of lung cancer.. Cancer Res.

[OCR_00651] Steinert P. M., Roop D. R. (1988). Molecular and cellular biology of intermediate filaments.. Annu Rev Biochem.

[OCR_00658] Stieber P., Bodenmüller H., Banauch D., Hasholzner U., Dessauer A., Ofenloch-Hähnle B., Jaworek D., Fateh-Moghadam A. (1993). Cytokeratin 19 fragments: a new marker for non-small-cell lung cancer.. Clin Biochem.

[OCR_00663] Stieber P., Hasholzner U., Bodenmüller H., Nagel D., Sunder-Plassmann L., Dienemann H., Meier W., Fateh-Moghadam A. (1993). CYFRA 21-1. A new marker in lung cancer.. Cancer.

[OCR_00670] Sugama Y., Kitamura S., Kawai T., Ohkubo A., Hasegawa S., Kuriyama T., Kato H., Fukuoka M., Ohkawa J. (1994). Clinical usefulness of CYFRA assay in diagnosing lung cancer: measurement of serum cytokeratin fragment.. Jpn J Cancer Res.

[OCR_00676] Sundström B. E., Nathrath W. B., Stigbrand T. I. (1989). Diversity in immunoreactivity of tumor-derived cytokeratin monoclonal antibodies.. J Histochem Cytochem.

[OCR_00679] Takada M., Masuda N., Matsuura E., Kusunoki Y., Matui K., Nakagawa K., Yana T., Tuyuguchi I., Oohata I., Fukuoka M. (1995). Measurement of cytokeratin 19 fragments as a marker of lung cancer by CYFRA 21-1 enzyme immunoassay.. Br J Cancer.

[OCR_00686] van der Gaast A., Schoenmakers C. H., Kok T. C., Blijenberg B. G., Cornillie F., Splinter T. A. (1994). Evaluation of a new tumour marker in patients with non-small-cell lung cancer: Cyfra 21.1.. Br J Cancer.

